# Effect of Control Strategies on Prevalence, Incidence and Re-infection of Clonorchiasis in Endemic Areas of China

**DOI:** 10.1371/journal.pntd.0000601

**Published:** 2010-02-16

**Authors:** Min-Ho Choi, Sue K. Park, Zhimin Li, Zhuo Ji, Gui Yu, Zheng Feng, Longqi Xu, Seung-Yull Cho, Han-Jong Rim, Soon-Hyung Lee, Sung-Tae Hong

**Affiliations:** 1 Department of Parasitology and Tropical Medicine and Institute of Endemic Diseases, Seoul National University College of Medicine, Seoul, Korea; 2 Department of Preventive Medicine, Seoul National University College of Medicine, Seoul, Korea; 3 Institute of Health Policy Management, Seoul National University, Seoul, Korea; 4 Heilongjiang Province Center for Disease Control and Prevention, Heilongjiang, China; 5 Zhaoyuan Center for Disease Control and Prevention, Heilongjiang, China; 6 National Institute of Parasitic Diseases, Chinese Center for Disease Control and Prevention, Shanghai, China; 7 Department of Molecular Parasitology, Sungkyunkwan University, Suwon, Korea; 8 Korea Association of Health Promotion, Seoul, Korea; Khon Kaen University, Thailand

## Abstract

**Background:**

A pilot clonorchiasis control project was implemented to evaluate the efficacies of various chemotherapy strategies on prevalence, incidence and re-infection in Heilongjiang Province, China.

**Methods and Findings:**

Seven intervention groups (14,139 residents, about 2000 in each group) in heavily or moderately endemic areas were subjected to repeated praziquantel administration from 2001 to 2004. In the selective chemotherapy groups, residents were examined for fecal eggs, and those who tested positive were treated with three doses of 25 mg/kg praziquantel at 5-hour-intervals in one day. However, all residents were treated in the mass chemotherapy groups. In heavily endemic areas, two mass treatments of all residents in 2001 and 2003 reduced the prevalence from 69.5% to 18.8%, while four annual mass treatments reduced the prevalence from 48.0% in 2001 to 8.4% in 2004. Selective annual treatments for egg-positive subjects reduced the egg-positive rates from 54.9% in 2001 to 15.0% in 2004 or from 73.2% in 2001 to 12.3% in 2004. Selective treatments every 6 months significantly reduced the prevalence from 59.5% in 2001 to 7.5% in 2004. All of the repeated treatments reduced EPG (eggs per gram of feces) significantly. The annual mass treatment and selective treatment every 6 months produced lower prevalence and re-infection rates and higher egg reduction rate than annual selective treatments did. In the moderate endemic areas, egg positive rates were 24.8% and 29.7% in 2001 but were 1.9% and 1.3% after 2 or 3 selective treatments. The prevalence, incidence, re-infection rates in a moderately endemic area were significantly lower than those of heavy endemic areas.

**Conclusions:**

Repeated mass treatment or selective treatment with praziquantel every 6 to 12 months is highly effective for clonorchiasis control in heavily endemic areas. In contrast, one or two selective treatments with health education is effective in moderately endemic areas.

## Author Summary

Clonorchiasis is a liver fluke disease prevalent in East Asia, which is transmitted to humans mainly by eating raw freshwater fish. It induces various complications in the liver or bile duct including cholelithiasis, cholecystitis, cholangitis, and cirrhosis. *Clonorchis sinensis* has been known to cause cholangiocarcinoma, and is still a major health problem in endemic areas. People in endemic areas are repeatedly infected with *C. sinensis*, as they continue to consume raw freshwater fish in spite of control activities and availability of a highly effective drug, praziquantel. Reservoir hosts such as cats, dogs, and pigs supply eggs continuously to the environment and act as a source of infection. The present study analyzed the data produced by the Korea-China collaborative project for helminthiasis control in China during 2001–2004 to find out effective chemotherapeutic control strategies with praziquantel in endemic areas and to evaluate their effects on the transmission of *C. sinensis* infection by repeated mass or selective treatment. The four-year control trial found that repeated treatment is essential to the effective reduction of prevalence and infection intensity in heavily endemic areas. Mass chemotherapy is more effective than selective treatment, and more repeated treatments produce better outcomes in clonorchiasis control. Health education to change the habit of consuming raw or undercooked fish is an important and practical measure to prevent and reduce human infections in endemic areas. Together with chemotherapy, health education could be highly effective and produce sustainable effects in clonorchiasis control. Treatment of reservoirs, if applicable, will contribute to reduce the source of infection.

## Introduction

*Clonorchis sinensis* is a liver fluke that infects humans and is widely prevalent in East Asia. An estimated 30 million people are infected by this fluke, and most of them are in China [1]. Since Heilongjiang Province has a wide plain region with many slow flowing rivers and branches, some of the wide river basin areas in the province, where local people eat raw fish, are suspected to be endemic of clonorchiasis [1],[2]. In surveys carried out from 1988 to 2002, the prevalence of clonorchiasis in human in Heilongjiang Province was 16.9% with the highest prevalence of 85.3%, and *C. sinensis* has been found highly prevalent in Korean (minority) communities in three northeastern provinces of China including Heilongjiang [1].

Although clonorchiasis is an inflammatory disease of the biliary tract, most cases are asymptomatic. Only a fraction of infected people suffer from subjective symptoms associated with complications of clonorchiasis, such as bile duct obstruction, stone formation, pyogenic cholangitis, abscess formation, and biliary cirrhosis [1],[3]. The most serious complication is cholangiocarcinoma, and high incidences of cholangiocarcinoma have been reported in endemic areas of clonorchiasis [4],[5]. Recently, *C. sinensis* has been classified as Group 1 biological agents that are carcinogenic to humans [6]. In heavy endemic areas without any intervention, the infection rate and infection intensity of clonorchiasis increase with age because of the accumulation of the long-living flukes and repeated infections. The accumulation effect by age showed an intensity peak in the 40 to 59 age group, and it became less severe after the age of 60 [3],[7]. This epidemiological phenomenon is interpreted that residents with clonorchiasis may die earlier due to cholangiocarcinoma or other complications than non-infected ones [3]. In case of *Opisthorchis viverrini*, the liver fluke in Thailand, incidence of cholangiocarcinoma was strongly associated with age, and persons aged 65–69 years showed 2.5 times higher incidence than other age groups [8].

When praziquantel was first introduced for treatment of *C. sinensis* infection in humans, it showed promise of successful control of clonorchiasis [9], but *C. sinensis* is still prevalent in endemic areas. There have been 3 trials of chemotherapeutic control with praziquantel [10],[11],[12]. The first trial was conducted in a small rural village with about 100 residents in Korea, and it revealed that repeated praziquantel treatment at 6-month-interval for 3.5 years was insufficient to achieve complete control of clonorchiasis [10]. The pilot study was carried out to investigate the efficacy of praziquantel and artemisinin on clonorchiasis in Vietnam, but poor cure rate (29%) of praziquantel has been reported because of inappropriate dosage, 25 mg/kg once daily for 3 days, instead of the conventional treatment regimen for clonorchiasis, 25 mg/kg, t.d. for one day [11]. The third trial carried out in two endemic villages in China showed that selective medication at 6-month-interval was more effective than 12-month-interval medication, but it was still incomplete for control of *C. sinensis* infection in heavy endemic areas [12].

The control strategies for clonorchiasis may differ according to the prevalence rates, infection intensity, resources available, and the compliance of the residents. The present study analyzed the data produced by the Korea-China collaborative project for helminthiasis control in China (KOICA project) in 2001–2004 to find out the optimum strategy of clonorchiasis control and to investigate the changes of incidence and re-infection rates after repeated chemotherapy in the study areas.

## Methods

### Ethics statement

The KOICA project has been implemented during the period of 2001–2004, which was financially supported by the Korea International Cooperation Agency (KOICA). The KOICA project comprised helminthiasis control activities in three provinces in China: control of clonorchiasis in Heilongjiang Province, that of soil-transmitted helminth infections in Jiangxi Province, and of food-borne parasite infections in Guangxi Province. The present study analyzed the data of clonorchiasis control in Heilongjiang Province produced by the KOICA project. The database was established from existing data and information in such a manner that all the subject information was well protected and could not be revealed directly or indirectly. In this regard, the research protocol has been exempted from IRB review of the Seoul National University College of Medicine in accordance with the exemption criteria (IRB No C-0905-007-279).

### Study areas and subjected population

**Figure 1:**
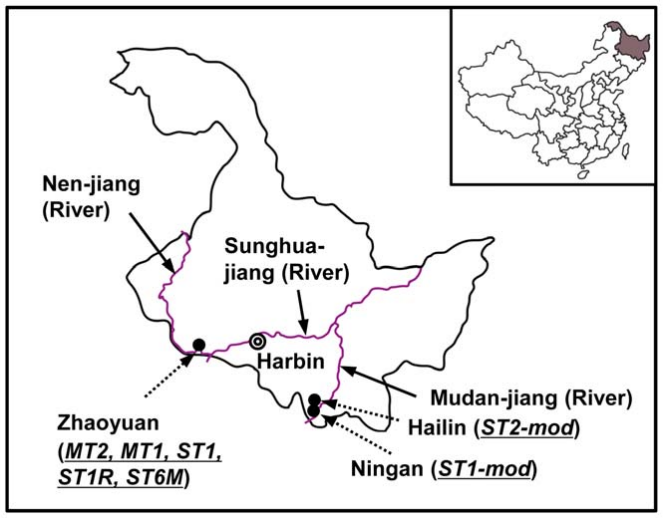
The map of the study areas in Heilongjiang Province, China. The study areas include five villages in heavy endemic areas of clonorchiasis in Zhaoyuan, and two villages in moderate endemic areas in Hailin and Ningan. MT2, mass treatment every 2 year; MT1, mass treatment every year; ST1, selective treatment every year; ST1R, selective treatment and reservoir control every year; ST6M, selective treatment every 6 month; ST2-mod, selective treatment every 2 year; ST1-mod, selective treatment every year.

Heilongjiang Province is located in the most northeastern area of China, bordering with Russia. It has a population of about 38 million, and the majority is Han Chinese with several ethnic minorities including Manchus, Koreans, and Mongols. It is the coldest province in China, and the annual rainfall ranges from 250 to 700 mm, mostly concentrated in June through August. The province has vast areas of flat agricultural land which is drained by several large rivers, including Sunghuajiang (jiang = river), Heilongjiang, Nenjiang, and Mudanjiang (Fig. 1). Most of those river basins provide good habitats for the intermediate hosts of *C. sinensis*, and species of cyprinid freshwater fish such as *Carassius auratus*, *Hemiculter leucisculus*, and *Pseudorasbora parva* act as the second intermediate hosts of *C. sinensis* [1].

The KOICA project was implemented in 7 villages in 3 endemic counties of Heilongjiang Province and was jointly conducted with the Heilongjiang Province Center for Disease Control and Prevention and the National Institute of Parasitic Diseases, Chinese Center for Disease Control and Prevention. Among 7 villages, 6 villages have a population of approximately 2,000 except for one village in Zhaoyuan (MT2 group) with a population of about 4,000. About 2,000 persons were enrolled in each intervention group. We analyzed the data from a total of 14,139 subjects (male 7,763 and female 6,376) between the ages of 2–86.

### Methods of examination and treatment

One stool sample was collected from each subject every 6 month, one or two years according to control strategies. Individual fecal samples were microscopically examined using the Kato-Katz (KK) method to detect and count eggs of *C. sinensis* [13]. Eggs per gram of feces (EPG) determined by the KK method were used as an index of intensity of infection: light (EPG 1–500), moderate (EPG 501–2,000), and heavy infection (EPG more than 2,001) [14]. Three doses of 25 mg/kg praziquantel (Distocide®, Shin Poong Pharmaceutical Co., Ltd., Seoul, Korea) at 5-hour-interval in one day were administered to all subjects to treat clonorchiasis [9]. Village doctors in the study areas actively participated in the distribution of praziquantel to the subjects and educated the people to take the drug according to the standard regimen for clonorchiasis.

### Strategy designs

**Figure 2:**
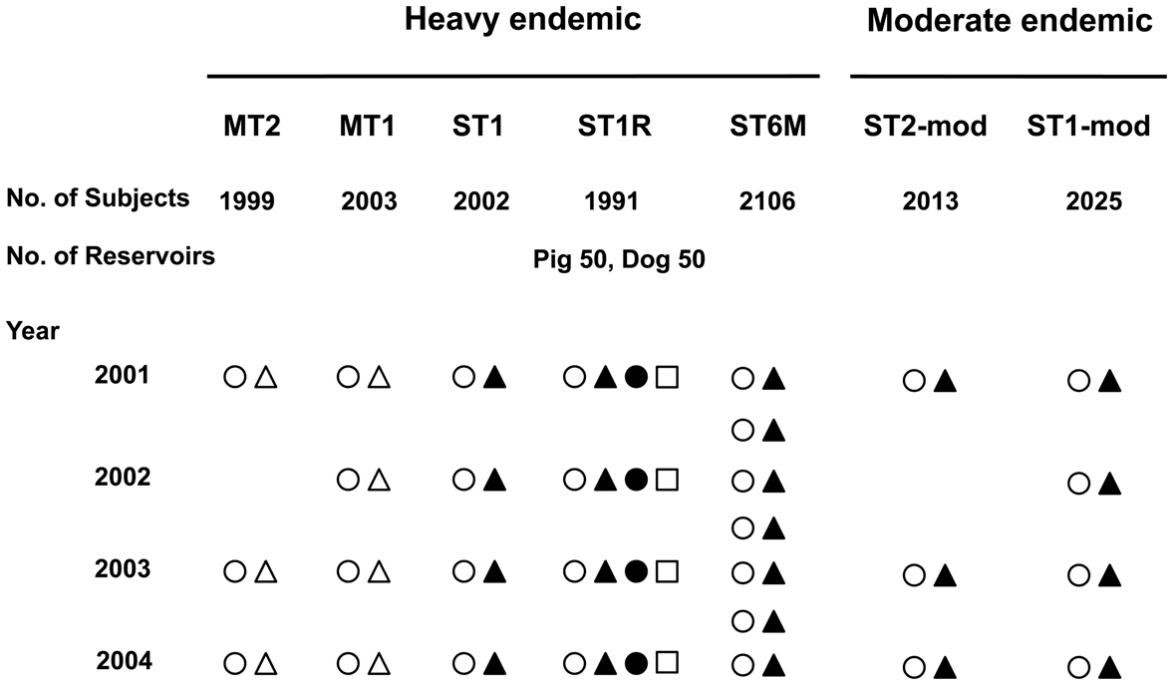
The schematic diagram of the Korea-China collaborative project for helminthiasis control in China (KOICA project), 2001–2004. ○, stool examination for human; •, stool examination for pigs and dogs; Δ, mass treatment for human; ▴, selective treatment for human; □, mass treatment for pigs and dogs. MT2, mass treatment every 2 year; MT1, mass treatment every year; ST1, selective treatment every year; ST1R, selective treatment and reservoir control every year; ST6M, selective treatment every 6 month; ST2-mod, selective treatment every 2 year; ST1-mod, selective treatment every year.

Different amounts of repeated medication were examined to determine the most efficient chemotherapeutic control with praziquantel (Table 1 and Fig. 2). Study areas were divided into two areas according to egg positive rates: moderate endemic areas with prevalence of 20-40% and heavy endemic areas with that higher than 40%. Five groups (villages) in Zhaoyuan County in heavy endemic areas and 2 groups in Hailin City and Ningan City in moderate endemic areas were selected (Figs. 1 and 2), and the enrolled persons have been followed up individually for their infection status. All of the subjects in the heavy endemic areas were selected for mass treatment (MT) and were treated regardless of fecal examination results. These were separated into the MT2 group (n = 1,999), which was treated every two years, and the MT1 group (n = 2,003), which was treated every year. In the 3 remaining heavy endemic groups, only fecal egg positive residents were treated as the selective treatment (ST) groups. These were separated into the ST1 group (n = 2,002), which was treated once every year, the ST1R group (n = 1,991), which was treated once every year as well as treatment of reservoir hosts, and the ST6M group (n = 2,106), which was treated once every 6 months. For the ST1R group, all reservoir hosts, including pigs and dogs, were treated with a single dose of 100 mg/kg praziquantel. The two groups in moderate endemic areas were also selective treatment groups and only fecal egg positive patients were treated. These were separated into the ST2-mod group (n = 2,013), which was treated every two years, and the ST1-mod group (n = 2,025), which was treated every year. Most of the residents in the heavy endemic were Han ethnic, while those of moderate endemic groups were Korean ethnic.

**Table 1:** Changes of egg positive rates of *Clonorchis sinensis* (Cs) by strategies, 2001-2004.

Endemicity	Treatment Strategies	No. of Subjects in 2001	Cs Prevalence Rates (PR, %) 1				POR (95% CI) 2	*p*-Value 3	Efficacy 4 of Each Strategy in Prevalence Control in 2004
			2001	2002	2003	2004			
**Heavy**	MT2	1999	69.5	— 6	33.2	18.8	1.2 (1.1–1.3)	<0.01	72.9
	MT1	2003	48.0	30.3	15.0	8.4	0.6 (0.5–0.7)	<0.01	82.5
	ST1	2002	54.9	36.1	20.0	15.0	1.0 (reference)	Reference	72.7
	ST1R	1991	73.2	47.8	16.7	12.3	0.7 (0.5–0.8)	<0.01	83.2
	ST6M 5	2106	59.5	29.9	19.2	7.5	0.4 (0.36–0.6)	<0.01	87.4
**Moderate**	ST2-mod	2013	24.8	- 6	7.1	1.9	0.1 (0.09–0.2)	<0.01	92.3
	ST1-mod	2025	29.7	1.8	2.4	1.3	0.1 (0.06–0.14)	<0.01	95.6

MT2, mass treatment every 2 year; MT1, mass treatment every year; ST1, selective treatment every year; ST1R, selective treatment and reservoir control every year; ST6M, selective treatment every 6 month; ST2-mod, selective treatment every 2 year; ST1-mod, selective treatment every year.

1 Cs prevalence rate (PR) for 1 year, weighted by age and sex of total population.

2 POR (95% CI) of each strategy relative to ST 1 year was computed by multivariate logistic model adjusted for age, sex and the prevalence in 2001.

3 *p*-value adjusted for age, sex and the prevalence in 2001 and corrected by Bonferroni's multiple comparison method.

4 Control efficacy = [PR in 2001 - PR in 2003 or 2004]/PR in 2001.

5 Mean prevalence rate for 1 year.

6 Not available.

### Statistical analysis of fecal examination data

The prevalence rate (PR) of *C. sinensis* infection was defined as the number of egg positive individuals detected per 100 participants. The PR with a 95% confidence interval (CI) was weighted by age group and sex according to the structure of the total study population. Control efficacy on prevalence was defined as [(PR in 2001 - PR in 2003 or 2004)/PR in 2001]. To compare the control efficacy on prevalence of each treatment strategy relative to that of the ST1 strategy, the prevalence odds ratios (PORs) and 95% CIs were calculated in multivariate logistic regression model, adjusted for age, sex, and infection intensity in 2001. Efficacy of each strategy on the reduction of egg counts among egg positive subjects was defined as the egg reduction rate (ERR) which was [(the geometric mean number of eggs in 2001 – the geometric mean number of eggs in 2003 or 2004)/(the geometric mean number of eggs in 2001)]. The incidence rate of new *C. sinensis* infection was defined as the number of newly infected residents from previously egg negative residents per 100 participants. The re-infection rate was defined as the number of positive changes from egg negative status after praziquantel treatment per 100 participants which were initially egg positive. To compare the incidence rates and re-infection rates of each treatment strategy in relation to the ST1 strategy, the relative risks (RRs) and 95% CIs of each treatment strategy were compared using Cox's proportional hazard model, and the RRs (95% CIs) of each treatment strategy were adjusted for age, sex, and infection intensity. To compare outcomes, such as prevalence, egg count, incidence, and re-infection in the seven intervention groups relative to the ST1 group, we calculated the *p*-values corrected by Bonferroni multiple comparison method [15]. We performed statistical analyses using SAS software for Windows version 9.2 (SAS Institute, NC, USA).

## Results

### Prevalence rates (PR) of *C. sinensis* infection by different strategies

A total of 14,139 persons were enrolled in the KOICA project, and each intervention group was composed of about 2,000 people. All the residents of the study areas were enrolled in the project except for MT2 group in Zhaoyuan County, where half of the residents were subjected for this study. The overall average compliance rate of enrolled subjects was 93.7% during the period of the study. The average yearly compliance rates of each intervention group were as follows: MT2 96.6%, MT1 88.7%, ST1 99.7%, ST1R 91.6%, ST6M 97.9%, ST2-mod 83.3%, and ST1-mod 98.4%, respectively.

Table 1 shows the changes of PRs of *C. sinensis* infection by each treatment strategy from 2001 to 2004. The PRs in the moderate endemic areas were significantly lower than those in the heavy endemic areas (*p*<0.01). The subjects of the ST1-mod group who lived in moderate endemic areas had better treatment result (POR = 0.1, 95% CI = 0.09–0.2 for ST2-mod; POR = 0.1, 95% CI = 0.06–0.14 for ST1-mod; *p*<0.01) than those of the ST1 group who lived in high endemic areas. In the moderate endemic areas, there was no difference between the ST2 and ST1 groups (*p*>0.05).

Among the groups in the heavy endemic areas, the PRs in the MT2 and MT1 groups were 18.8% and 8.4% in 2004, respectively. Relative to the reference ST1 group, the MT1 group had a 0.6-fold lower PR (15.0% for ST1 group; 8.4% for MT1 group; POR = 0.6, 95% CI = 0.5–0.7; *p*<0.01), while the MT2 group had a higher PR (18.8%) than the ST1 group (POR = 1.2, 95% CI = 1.1–1.3, *p*<0.01). In the 3 groups of ST strategy, the groups with more frequent treatments (ST6M and ST1R) had 0.4-fold and 0.7-fold lower POR, respectively, than the ST1 group (both *p*<0.01).

### Egg reduction rates (ERR) by different strategies among *C. sinensis*-infected populations

**Figure 3:**
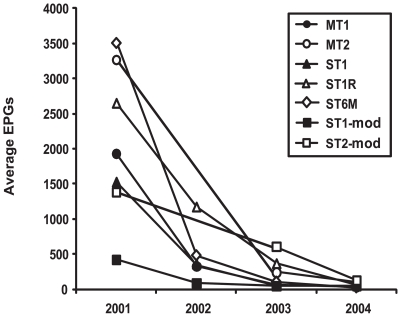
Egg reduction by different control strategies. Average number of eggs per gram of feces (EPG) of each group was used to evaluate the efficacy of control strategies. The EPG counts decreased dramatically during the study period in both heavy and moderate endemic areas. MT2, mass treatment every 2 year; MT1, mass treatment every year; ST1, selective treatment every year; ST1R, selective treatment and reservoir control every year; ST6M, selective treatment every 6 month; ST2-mod, selective treatment every 2 year; ST1-mod, selective treatment every year.

At the beginning of this project, the proportions of residents with heavy and moderate infections were 21.1% and 22.8%, respectively. Heavily infected residents were not identified after interventions and all of the egg positive residents were lightly infected. Population-based ERRs were between 89.1% and 99.4% from 2001 to 2004 (Fig. 3). Although the EPG counts decreased during the study period in both heavy and moderate endemic areas, the population-based ERRs in moderate endemic areas were significantly lower than those in heavy endemic areas (*p*<0.01). Among the infected subjects in the heavy endemic areas, the ERRs in the MT1 and ST6M groups were higher than those of the ST1 group (both *p*<0.01), but those in the MT2 and ST1R groups were not significantly different.

### Prevalence by gender and age

More males were infected with *C. sinensis* than females in 2001 (2,742 male and 1,880 female in 2001; *p*<0.001). However, no significant difference was found between genders in 2002–2004 (657 male and 618 female in 2002, *p* = 0.6; 213 male and 178 female in 2003, *p* = 0.5; 65 male and 67 female in 2004, *p* = 0.7).

After age stratification, the control efficacy between 2001 and 2004 in moderate endemic areas in all age groups was significantly higher than that in heavy endemic areas (*p*<0.001 in all age groups) (data not shown). In heavy endemic areas, there is no significant difference between the MT2 and ST1 group in control efficacy irrespective of age (*p*>0.05) (Table 2). The control efficacy among the subjects under age 20 in ST1R and ST6M was higher than that of ST1 (*p*<0.05), whereas MT2 and MT1 groups showed no difference in the control efficacy with ST1 group (*p*>0.05). In contrast, among the subjects over and at age 20, the control efficacy of each strategy was significantly different from that of ST1 group (the all *p*<0.05) and the control efficacy was ranked as below: the highest in ST6M, followed by ST1R, MT1, ST1 and MT2 in decreasing order (Table 2).

**Table 2:** Control efficacy on prevalence rate in the study areas according to age and treatment strategy.

Endemicity	Treatment Strategies	Age	No. of Subjects Cs-Tested in 2003	Cs Prevalence Rates in 2001 (%) 1	Cs Prevalence Rates in 2004 (%) 1	Control Efficacy between 2001 and 2004	*p*-Value 2
**Heavy**			9346	58.3	18.8	67.8	
	MT2	Total	1938	69.5	18.8	72.9	
		−19	226	50.4	17.8	64.7	0.09
		20–39	916	73.9	20.2	72.7	0.09
		40–59	707	69.0	17.4	73.8	0.45
		60+	89	64.5	18.2	71.8	0.10
	MT1	Total	1745	48.0	8.4	82.5	
		−19	424	31.7	9.9	68.8	0.99
		20–39	709	45.6	8.7	80.9	<0.01
		40–59	547	52.6	8.1	84.6	<0.01
		60+	65	54.9	6.4	88.3	0.02
	ST1	Total	1778	54.9	15.0	72.7	
		−19	424	30.0	9.6	68.0	reference
		20–39	723	53.2	14.8	72.2	reference
		40–59	541	61.7	15.2	75.4	reference
		60+	90	54.1	20.5	62.1	reference
	ST1R	Total	1825	73.2	12.3	83.2	
		−19	298	46.9	9.9	78.9	0.049
		20–39	811	71.5	11.1	84.5	<0.01
		40–59	646	78.5	14.4	81.7	0.045
		60+	70	70.3	4.5	93.6	<0.01
	ST6M	Total	2060	59.5	7.5	87.4	
		−19	273	43.0	5.8	86.5	0.02
		20–39	907	57.6	6.4	88.9	<0.01
		40–59	763	62.0	7.5	87.9	<0.01
		60+	117	65.0	9.4	86.6	0.02
**Moderate**	ST1-mod or ST2-mod	Total	3511	26.0	1.4	94.6	
		−19	358	4.7	0	100	
		20–39	1417	20.0	1.1	94.5	
		40–59	1522	35.3	2.2	93.8	
		60+	214	19.7	0.5	97.5	

MT2, mass treatment every 2 year; MT1, mass treatment every year; ST1, selective treatment every year; ST1R, selective treatment and reservoir control every year; ST6M, selective treatment every 6 month; ST2-mod, selective treatment every 2 year; ST1-mod, selective treatment every year.

1 Age specific CS prevalence rate (PR) for 1 year.

2 *p*-value for group difference among the specific age groups with each strategy in heavy endemic areas.

### Incidence

Table 3 presents incidence rates (from egg negative in 2001 to egg positive by 2004) in each intervention group. Incidence depended mainly upon the endemicity in the area. Even though the same strategy of ST was used, subjects living in the high endemic areas showed higher incidence rates than those living in the moderate endemic areas (RR = 0.2, 95% CI = 0.1–0.3 for ST2-mod; RR = 0.1, 95% CI = 0.1–0.3 for ST1-mod). In the high endemic areas, the MT2 group showed a higher incidence rate than the ST1 group (RR = 1.7, 95% CI = 1.2–2.5, *p* = 0.027), while the incidence rate in the groups with other strategies was not statistically different from that in the ST1 group.

**Table 3:** New incidence rates from egg negative to egg positive status by treatment strategies among initial negative subjects for *Clonorchis sinensis* (Cs), 2001–2004.

Endemicity	Treatment Strategies	No of Cs Negative Subjects in 2001	Incidences of New Cs per 100 Persons			RR (95% CI) 1	*p*-Value 2
			2002	2003	2004		
**Heavy**	MT2	1445	— 4	20.4	10.8	1.7 (1.2–2.5)	0.027
	MT1	1099	0.8	8.8	7.6	1.2 (0.8–1.7)	1.0
	ST1	866	0.5	4.4	6.4	1.0 (reference)	Reference
	ST1R	542	7.8	5.7	4.2	0.7 (0.4–1.1)	0.7
	ST6M 3	866	4.7	6.0	5.8	0.8 (0.5–1.2)	1.0
**Moderate**	ST2-mod	1099	— 4	3.9	0.9	0.2 (0.1–0.3)	<0.01
	ST1-mod	1445	0	0.8	1.0	0.1 (0.1–0.3)	<0.01

MT2, mass treatment every 2 year; MT1, mass treatment every year; ST1, selective treatment every year; ST1R, selective treatment and reservoir control every year; ST6M, selective treatment every 6 month; ST2-mod, selective treatment every 2 year; ST1-mod, selective treatment every year.

1 The relative risk (RR) and confidence interval (CI) were estimated by Cox's proportional hazard model, adjusted for age and sex.

2 Age and sex adjusted *p*-values corrected by Bonferroni's multiple comparison method.

3 Mean numbers of every year.

4 Not available.

### Re-infection rate

Some subjects, who were egg positive in 2001 and egg negative in 2002 after praziquantel therapy, became egg positive again at a proportion from 4.1% to 9.2% in 2003 and 0.7% to 12.5% in 2004 (Table 4). The re-infection rates in the moderate endemic areas were significantly lower than in the heavy endemic areas (*p*<0.01). The re-infection rate was significantly lower in the MT1 and MT2 strategy groups than in the ST1 strategy group (RR = 0.4, 95% CI = 0.2–0.8, *p* = 0.04 for MT1; RR = 0.4, 95% CI = 0.3–0.7, *p*<0.01 for MT2). The ST6M group, which was subjected to more frequent praziquantel treatments, showed a lower re-infection rate than the ST1 group (RR = 0.4, 95% CI = 0.2–0.6, *p*<0.01). The re-infection rates of the ST1R and the ST1 groups did not differ (*p* = 1.0) even though the reservoir host control was implemented simultaneously with treatment of infected persons.

**Table 4:** Re-infection rates changes from *Clonorchis sinensis* (Cs) egg positive to negative status by treatment and egg positive again by treatment strategies among initial Cs positive subjects, 2001–2004.

Endemicity	Treatment Strategies	No. of Cs Egg Negative Changed First Treatment	Recurrence Rates (%)		RR (95% CI) 1	*p*-Value 2
			2003	2004		
**Heavy**	MT2	782	— 4	5.1	0.4 (0.3–0.7)	<0.01
	MT1	322	7.0	5.2	0.4 (0.2–0.8)	0.04
	ST1	348	9.2	12.5	1.0 (reference)	Reference
	ST1R	487	4.2	10.8	0.8 (0.5–1.2)	1.0
	ST6M 3	639	7.4	4.3	0.4 (0.2–0.6)	<0.01
**Moderate**	ST2-mod	279	— 4	0.7	0.1 (0.01–0.2)	<0.01
	ST1-mod	514	4.1	1.6	0.1 (0.06–0.3)	<0.01

MT2, mass treatment every 2 year; MT1, mass treatment every year; ST1, selective treatment every year; ST1R, selective treatment and reservoir control every year; ST6M, selective treatment every 6 month; ST2-mod, selective treatment every 2 year; ST1-mod, selective treatment every year.

1 The relative risk (RR) and 95% confidence interval (CI) in 2004 recurrence rates were estimated by Cox's proportional hazard model, adjusted for age and sex.

2 Age and sex adjusted *p*-value corrected by Bonferroni's multiple comparison method.

3 Mean numbers for each year.

4 Not available.

### Control of reservoir hosts

About 1,000 pigs and 300 dogs were raised in the villages of the ST1R group, and all were treated once a year. Fifty of each animal were sampled for fecal examination to evaluate the prevalence of *C. sinensis* infection among reservoirs. Twenty-seven (54%) of 50 pigs and 29 (58%) of 50 dogs were egg positive in 2002, but the positive rates decreased to 24% of pigs and 10% of dogs in 2003.

## Discussion

The findings from this study prove that the selective treatment (ST) with praziquantel is effective in moderate endemic areas but that repeated treatment is required in heavy endemic areas to control *C. sinensis* infection. Three repeated annual MT and ST every 6- or 12-month are effective in reducing not only the prevalence but also intensity of infection, incidence and re-infection rate. In heavy endemic areas, MT strategy is recommended based on its high efficacy. By contrast, the control was very effective in the moderate endemic areas regardless of medication frequency. Most of the cured residents in the moderate endemic areas were not re-infected, in contrast to the ones in the heavy endemic areas. Therefore, one or two ST plus health education is highly effective in the areas of moderate endemicity.

Our study confirmed that control efficacy of clonorchiasis is correlated with the frequency of praziquantel administration (Table 1 & Fig. 3). Prevalence rates decreased in all 7 groups, and the 6-month interval ST group showed the best control efficacy, with 0.4 POR (95% CI 0.36–0.6) and 99.4% ERR. In this ST6M group, the egg positive residents received a total 7 administrations of praziquantel at an interval of 6 months during the project period. Although this strategy produced the highest control efficacy, it may not be practical in the field because of cost-effectiveness as it requires too much labor and expense for laboratory work and the drug.

The ST1 group was the standard for group comparison, and its POR was assumed to be 1. The efficacy of ST1 was 72.7%. The efficacy of another 1-year ST group with reservoir host control (ST1R) was 83.2%, and the 0.7 POR value (95% CI 0.5–0.8) was significantly different from the ST1 group. The MT1 group had an efficacy of 82.5% and a POR of 0.6 (95% CI 0.5–0.7), which was a significant improvement. Based on these findings, MT is more effective than ST for controlling heavy endemic clonorchiasis on a large scale.

In the heavy endemic areas, approximately half of the residents were infected, and the MT included praziquantel therapy of the remaining uninfected half. Hypothetically, therefore, MT should be a better control strategy than ST because it may cover the undiscovered infected cases due to the limited sensitivity of the KK method or other fecal examination methods [13],[16]. Therefore, it is important to treat those hidden cases in a control program. Furthermore, MT is recommended in heavy endemicity zones even when cost-effectiveness is considered because MT may reduce the cost of fecal examination for all subjects. In Africa, an integrated MT program is recommended with continuous monitoring of sampled population for control of major neglected tropical diseases on the basis of calculating cost-effectiveness [17].

Our results clearly demonstrate that any single chemotherapy with praziquantel administered either by MT or ST is insufficient to control clonorchiasis, and that repeated annual chemotherapy is essential in the heavy endemic areas where egg positive rate is over 40%. In most of the study groups, single chemotherapy of MT or ST reduced the egg positive rates to only half of the original ones, although the cure rate of praziquantel treatment is as high as 80–85% after one month [3],[9]. This low cure rate following 6-month or 1-year treatment regimens arose from either of re-infection after cure or incomplete cure [10],[12].

Re-infection or super-infection is common in endemic areas and may be the main cause of consistent transmission in the endemic areas. For example, the egg positive rate in the ST1R group rebounded to 20.2% in 2006, 2 years after the annual ST in 2004 when the egg positive rate was 12.3%. Re-infection still remained high although annual ST had been repeated 4 times in this village. Because the residents prefer eating raw fish and their food habit is difficult to change within a short time of period, re-infection occurs consistently, which is a crucial obstacle in the control of clonorchiasis. Another possible factor of the low cure rate is the pharmacokinetic nature of praziquantel. A total dose of praziquantel divided into 3 portions taken at 5-hour-interval may reach a cure rate of 80% confined by its pharmacokinetics [5],[9]. However, the second or third dose is often forgotten or delayed which lowers cure rates but that is inevitable in any large scale control program.

The data were analyzed to estimate the incidence of egg negative and re-infection status of cured subjects. The incidence and re-infection suggested strong transmission intensity present in the study areas. The incidence rates were between 4.2 and 10.8 in 2004 in heavy endemic areas while those were 0.9 and 1.0 in moderate endemic areas. The incidence was relatively irrelevant with the repeated praziquantel therapy groups but closely related with the endemicity, which determined the transmission intensity at the individual village. This intensity of clonorchiasis in endemic areas is determined by the transmissibility of *C. sinensis* eggs in night soil and the metacercaria in the intermediate hosts present in a much wider area around the project villages. Recently the incidence of opisthorchiasis was reported 21.6/100 person-years in a Thai endemic village [18]. Compared with the incidence of opisthorchiasis, the present incidence rates of clonorchiasis were not so high in Heilongjiang province, China. The incidence of the liver fluke disease may vary individually according to the subjected villages.

The re-infection of the cured subjects in 2004 ranged from 4.3% in the ST6M group to 12.5% in the ST1 group in the heavy endemic areas, while the rates were 0.7% and 1.6%, respectively, in the moderate endemic areas. The relative risks were significantly lower in the MT2 and ST6M groups in the heavy endemic areas and in all groups in moderate endemic areas.

In an endemic area of opisthorchiasis in Thailand, people with high intensities of infection before treatment showed a tendency of higher re-infection rates and of heavier intensities of re-infection than those with light infection or egg-negative residents [19]. This finding suggested that some people may be predisposed to rapid re-infections with moderate and heavy infections, probably due to environmental and individual's genetic or behavioral factors [19]. However, there was no predisposition to heavy infections among inhabitants with high pre-treatment intensities of infection in this study. Unequal predisposition to infection suggests that chemotherapy should be targeted preferentially to individuals with a predisposing tendency to *O. viveriini* infection, but in contrast, control interventions for clonorchiasis should be applied to the entire population or infected residents regardless of intensities of infection of individuals.

The ST1R group included praziquantel therapy of all of pigs and dogs which lived together with residents in the village. Hypothetically, the reservoir hosts are important for control efficacy because they are a potential source of eggs which are spread in the environment [17]. Therefore, even after human hosts are completely cured from clonorchiasis, transmission may continue due to the eggs spread by the reservoir hosts. Half of the pigs and dogs examined were infected, and most of them were cured after the annual treatment. Their cure rate was better than that of residents because they were treated with higher dose of praziquantel (100 mg/kg) than humans. The control of the reservoir hosts produced a higher efficacy but it was too laborious. Control intervention should be carried out in an area to cover the entire human population and reservoir hosts as far as possible in a specific river basin to interrupt the life cycle of *C. sinensis*.

Changes in eating behavior through health education may contribute most effectively to the control. In addition to the chemotherapy, health education was implemented to supplement the control activities in the KOICA project. The education combined special teaching programs on TV and radio, demonstration on billboards, propaganda painting, educational video CDs, distribution of booklets, and slogans on building walls to educate residents of the pilot villages. The residents were encouraged to change their habit of eating raw fish and to elevate their health care knowledge. In the field, the practice of feces collection and distribution of the drug may play a role of education. Moreover, the village doctors delivered praziquantel tablets to the subjects individually, and at the same time, performed health education to consolidate the achievement of control.

Although the effectiveness of health education was not evaluated in this KOICA project, chemotherapy and health education may produce a synergistic outcome in clonorchiasis control. During the control activities in this project, we found that people in the study areas became well aware of the fish-borne transmission of *C. sinensis*, and they actively participated in the stool examination and praziquantel treatment. After the termination of this project, health personnel in Zhaoyuan Center for Disease Control and Prevention (CDC) successfully got the financial support from their local government, and performed the control activity for clonorchiasis by themselves using the control strategy of the KOICA project.

The side effects of praziquantel include headache, dizziness, nausea, vomiting, diarrhea, abdominal pain, and rash [3]. These adverse reactions are usually mild, transient, and spontaneously disappear without special treatment. So far, three cases of anaphylactic reactions to praziquantel have been reported [20], and albendazole could be an alternative for anaphylactic reaction to praziquantel. Although praziquantel is generally regarded as safe, doctors and field workers should be aware of the possible adverse effects of praziquantel.

The KK method has been found sensitive and reliable to diagnose clonorchiasis, except for extremely light infections with EPGs less than 24 [13]. It is widely used especially in the field because of its simple, rapid, and cheap characteristics. Furthermore, both diagnosis and intensity of infection can be determined at the same time by this method. In study areas, very low prevalence of parasitic infections other than *C. sinensis* infection was observed: ascariasis (0.23%), trichuriasis (0.22%), hookworm infection (0.007%), and *Schistosoma japonicum* infection (0.05%). No infection with intestinal trematode was noted, and therefore, there was no difficulty in differential diagnosis between *C. sinensis* and other minute intestinal flukes using the KK method.

Clonorchiasis is prevalent at a moderate to heavy endemicity along the Sunghuajiang (river) and its branches in Heilongjiang Province. It was estimated that the endemic areas may be widely distributed in a large area along the riverside and in the areas having aquaculture ponds in the province. Although control efforts have been made, the data recorded in this study indicated that clonorchiasis remained highly prevalent in some parts of Heilongjiang Province, where the local people have the habit of eating raw fish. Evidently, a persistent intervention program with wider coverage of endemic areas is necessary to bring clonorchiasis under control.

In conclusion, we suggest that repeated annual mass treatment or selective treatment with praziquantel is effective for clonorchiasis control in heavy endemic areas. In moderate endemic areas, one or two ST combined with health education is highly effective. Chemotherapy combined with health education is more effective and produce long-lasting control effect than chemotherapy alone. Furthermore, the education programs to avoid consuming raw or undercooked freshwater fish should be particularly focused on young children, because they are more likely to change their food habits than the older population. Reservoir control, if applicable, will contribute to reduce the source of infection.
